# Preparation and characterization of thermo-sensitive gel with phenolated alkali lignin

**DOI:** 10.1038/s41598-018-32672-z

**Published:** 2018-09-27

**Authors:** Pan Jiang, Xueru Sheng, Sheng Yu, Haiming Li, Jie Lu, Jinghui Zhou, Haisong Wang

**Affiliations:** College of Light Industry and Chemical Engineering, Dalian Polytechnic University, Dalian, Liaoning 116034 China

## Abstract

Thermo-sensitive gel exhibits great potential industrial application. It has been widely used in tissue repair, drug release and water purification for its property of phase transition in response to external stimuli, reusability and biocompatibility. In this study, a novel lignin-based thermo-sensitive gel was synthesized with alkali lignin by two steps. Firstly, phenolated lignin (PPAL) was synthesized with purified alkali lignin (PAL) catalyzed by sulfuric acid. Subsequently, thermo-sensitive gel was achieved by thermal polymerization of phenolated alkali lignin and N-isopropylacrylamide (NIPAAm). Furthermore, the prepared hydrogels were characterized in terms of thermal behavior, interior morphology and their swelling behavior. Compared with PAL-based gel, the obtained PPAL-based gel exhibits a higher crosslinking density and lower critical solution temperature (LCST) due to the increase of reaction site and smaller space volume of the hydrophobic side groups grafted on NIPAAm. TGA data revealed that thermal stability of gel was enhanced (50% weight loss at ~380 °C) by using lignin as precursor. SEM images showed that a more regular interior morphology, better compressive strength was also found (PPAL0.05, 11.15 KPa). Furthermore, the swelling ratio of PPAL-based gel was lower than that of PAL-based gel due to its more complex structure.

## Introduction

With the depletion of fossil energy and the development of polymer industry, bio-renewable polymers have attracted a greater attention. On one hand, lignin is the only bio-polymer which is composed of aromatics. It has been considered as an ideal substitute for polymer production^1–4^. On the other hand, smart hydrogel has the ability that undergo reversible phase transition in response to external stimuli (*e*.*g*. pH^5^, temperature^6,7^, CO_2_^8^, glucose concentration^9^) and has been widely used in tissue repair^10–12^, drug release^13–15^, biosensing^16,17^ and waste water treatment^18,19^. Several attempts have been made to synthetize lignin-based thermo-sensitive hydrogel by grafting N-isopropyl acrylamide (NIPAAm) monomer onto lignin, it shows great reusability and biocompatibility^3,20–22^. However, the application of lignin in lignin-based thermo-sensitive hydrogel formation is limited by its low purity, complex structure and poor reaction activity^23^. Increasing the content of reactive functional groups such as hydroxyl group in lignin through chemical modification can effectively overcome this shortcoming, and it is expected that the modified lignin-based thermo-sensitive hydrogel will be more widely used in various fields. For biomedical application, there are some concerns regarding the potential cytotoxicity of lignin. In fact, several types of industrial lignin have been tested for its cell cytotoxicity, the result shows that lignin is safe to cell and relative products are also not cytotoxic, it could be incorporated into cells^24–26^. These studies resolved the doubts regarding the safety of lignin for potential applications in cosmetics and pharmaceuticals. Moreover, the three-dimensional structure of lignin and gel can provides more drug loading sites and loading capacities. On top of this, lignin-based thermo-sensitive hydrogel is an ideal candidate for highly effective stimuli-responsive drug delivery carriers with high drug loading capacities. For agricultural application, this gel, with LCST around 32 °C which is close to natural temperature, is expected to apply in agricultural field as a pesticide carrier with property of stimuli release and absorption based on the change of natural environment temperature^27^.

In this work, it was first reported that a novel lignin-based thermo-sensitive hydrogel was synthesized with phenolated lignin (PPAL) and the obtained hydrogels were characterized by different methods. For the synthesis step, purified wheat alkali lignin (PAL) was modified with phenol in order to improve the hydroxy content of lignin, which can promote the reaction activity and grafting rate in the subsequent polymerization. Moreover, the properties of gels were also enhanced by the increasement of crosslinking density. For the characterization step, the swelling behavior, thermal property, interior morphology, mechanical property of obtained lignin-based gels were also investigated.

## Results and Discussion

**Figure 1:**
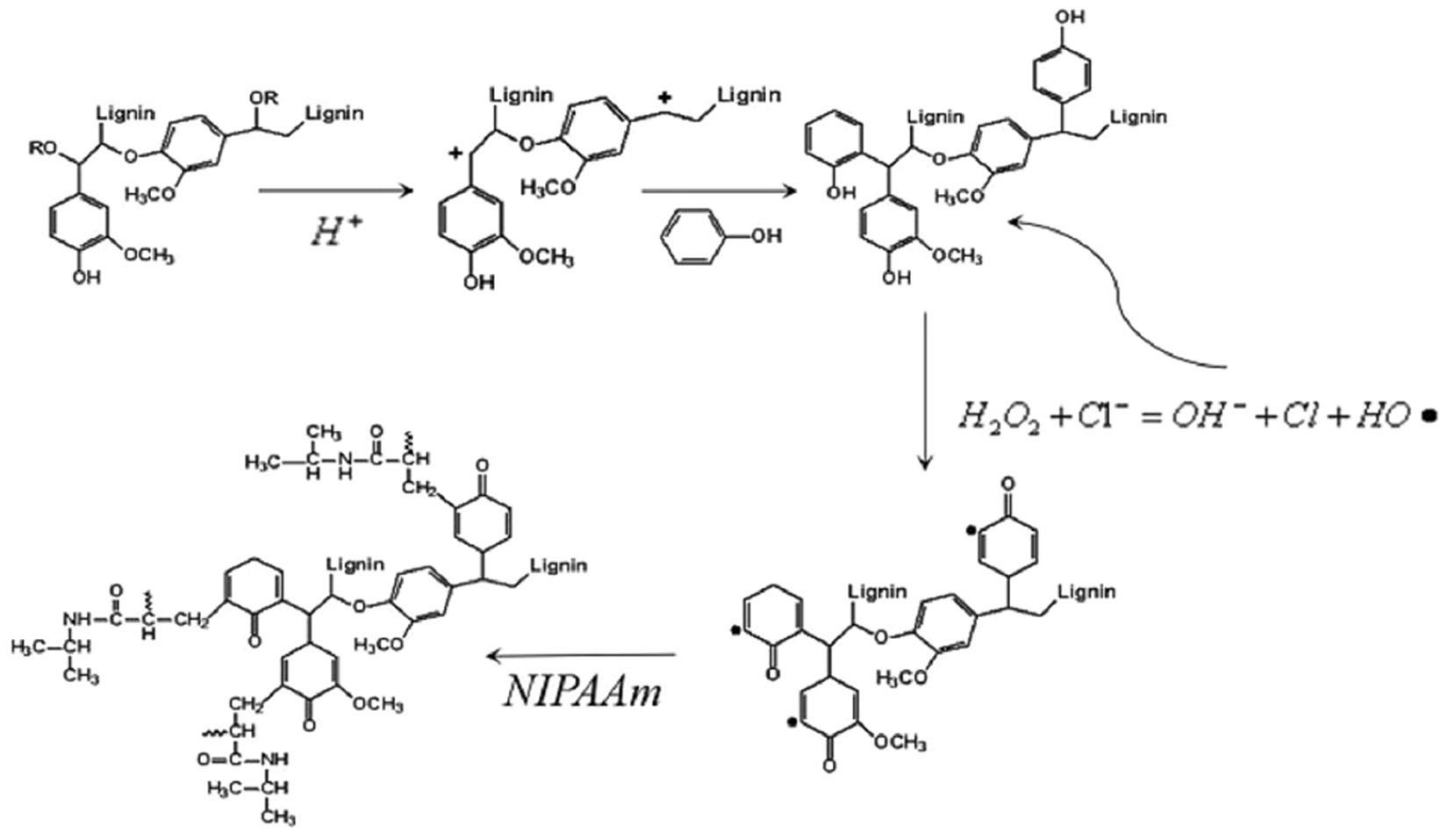
Synthetic scheme for the preparation of PPAL-based gel.

PPAL-based gel was synthesized via two steps: (i) the preparation of PPAL. (ii) the polymerization of PPAL and NIPAAm (as shown in Fig. 1).

### Synthesis and characterization of PPAL

**Figure 2:**
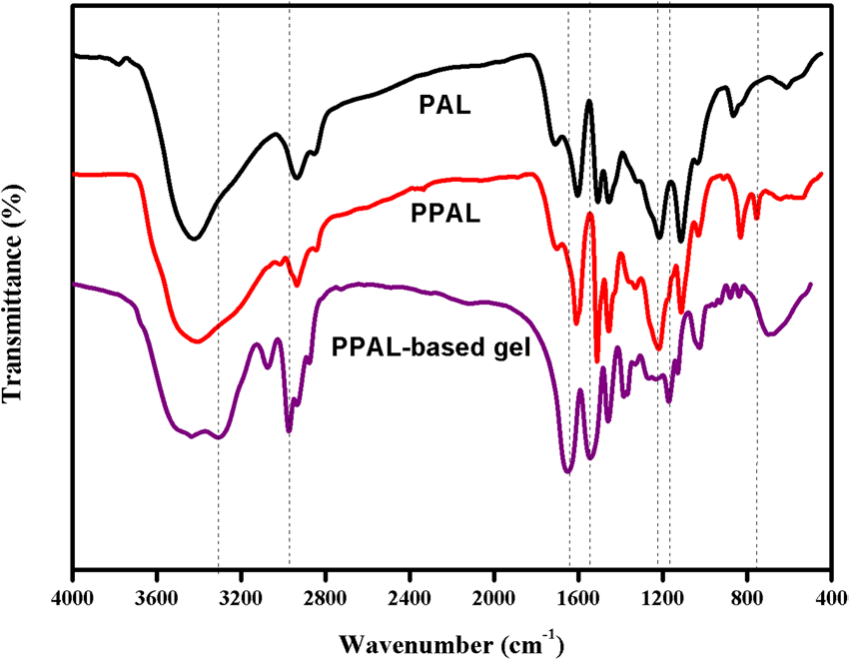
FT-IR spectra of PAL, PPAL and lignin-based gel.

PPAL was synthesized with PAL according to the steps shown in methods. Afterwards, FT-IR, GPC, H-NMR were used to investigate the effect of phenol modification. Firstly, FT-IR was carried out to investigate the functional groups on PAL and PPAL. As shown in Fig. 2, PAL possessed the characteristic peaks at 3429, 2935, 1604/1503 and 863 cm^−1^, which were attributed to the -OH stretching vibration, C-H stretching vibration of methyl (or methylene), skeleton vibration of aromatic rings and C-H out-plane flexural vibration on aromatic rings, respectively. Comparing to PAL, the characteristic peak on PPAL varied as below: (i) -OH stretching vibration at 3430 cm^−1^ was broadened; (ii) C-O stretching vibration at 1224 cm^−1^ was more visible compared with that in PAL; (iii) a new characteristic peak occurred at 749 cm^−1^ which was attributed to the C-H out-plane flexural vibration on aromatic rings. Above-mentioned indicates that condensation of phenol with lignin has occurred. After grafting reaction, the hydrogels prepared from PAL and PPAL showed the same infrared information about NIPAAm. The characteristic peaks were attributed to N-H stretching vibration, C-H stretching vibration of methyl or methylene, C=O stretching vibration, C-N stretching vibration on NIPAAm appeared at 3303/1554, 2941, 1648, 1167 cm^−1^, respectively. The C-O stretching vibration at 1224 cm^−1^ was weakened due to graft copolymerization.

**Figure 3:**
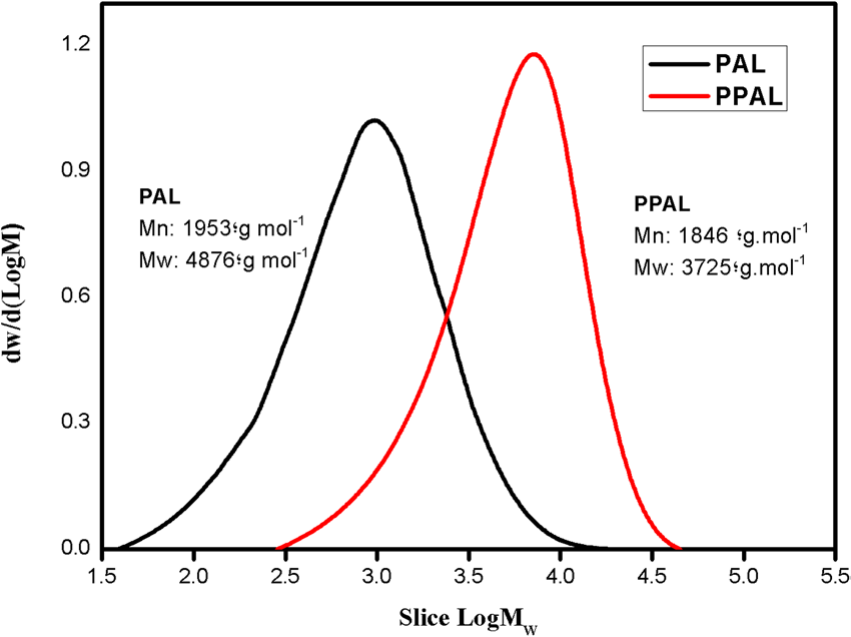
GPC chromatograms of PAL and PPAL.

Subsequently, GPC analysis was performed to investigate the molecular weights of lignin after phenolation. As shown in Fig. 3, number-average molecular weight (M_n_) and weight-average molecular weight (M_w_) of PPAL were both decreased (from 1953,4876 g/mol to 1846, 3725 g/mol, respectively). As we know, lignin is a three-dimensional natural macromolecule connected by three types of unit (G, H, S) through ether bond, C-C bond, in which α-O-4 linkage occupies 2–8% of the total chemical bond^28^. Since strong mineral acid was used during phenolation, the α-O-4 linkage cleaved and formed cationic active site on the α-C of lignin side chain (as shown in Fig. 1). Phenol, the nucleophilic reagent, was grafted onto the lignin structure, preferentially and preventing the condensation reaction of lignin occurred at alpha position during phenolation, thus resulted in partially degradation of PAL, expressing in the reduction of molecular weight.

**Figure 4:**
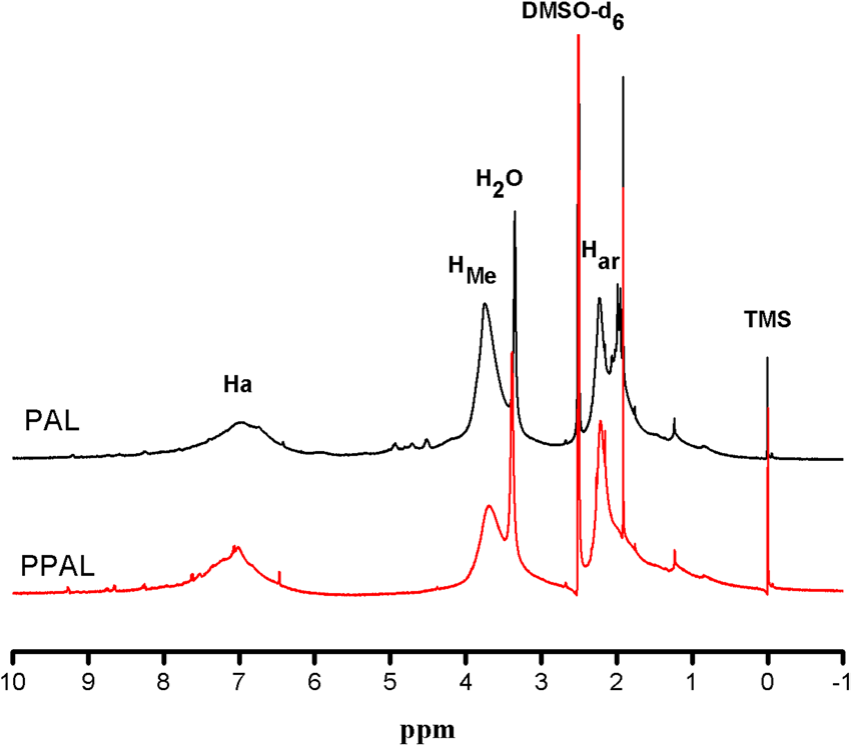
H-NMR spectra of PAL, PPAL.

Figure 4 shows hydrogen protons change during phenol modification. Peaks at 6.0–8.0, 3.5–4.0, 2.0–2.4 ppm belong to aromatic protons, methoxy protons, phenolic protons (acetylation), respectively. It could be observed that the intensity of aromatic protons, phenolic protons (acetylation) in PPAL increased compared with PAL, implying that the phenol was successfully grafted onto lignin. Furthermore, the peak corresponding to methoxy protons decreased due to demethoxy reaction during phenolation.

**Figure 5:**
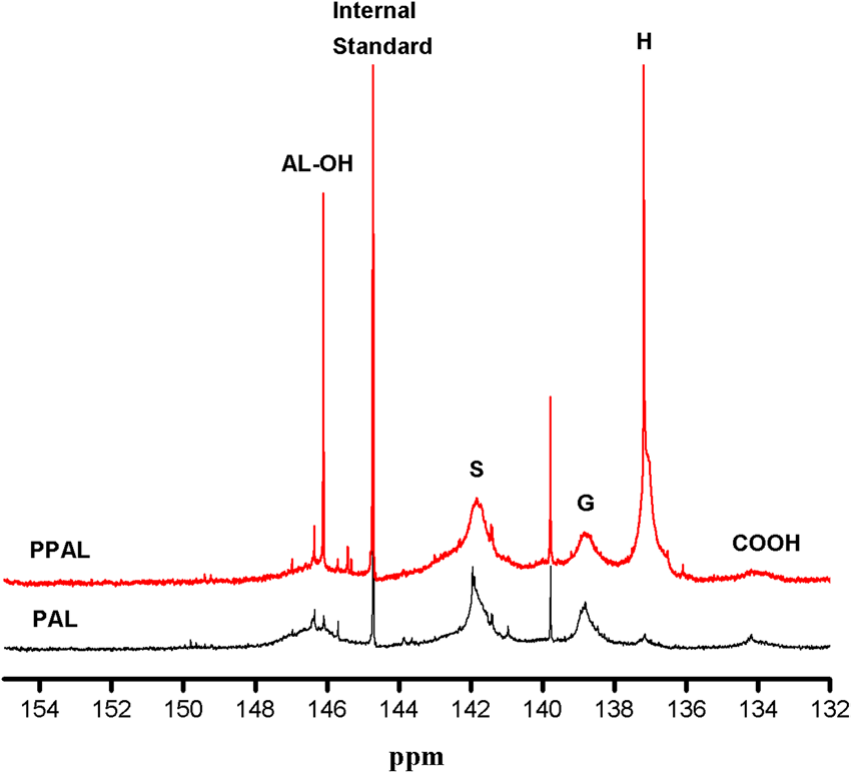
^31^P-NMR spectra of PAL, PPAL.

According to the literature, the low polymerization activity of lignin was due to high substitution on benzene rings and the steric hindrance caused by its complex molecular structure^29^. Therefore, the content of several types hydroxyl groups in PAL and PPAL was also investigated by ^31^P-NMR (as shown in Fig. 5 and Table 1). In detail, aliphatic OH was located at the range of 145–148 ppm, whereas three types of aromatic OH including syringyl OH, guaiacyl OH, *p*-hydroxy phenyl OH were range from 141.5-143, 138.5-139.5, 137-137.9 ppm, respectively. In addition, the carboxylic OH was calculated on the basis of the range of 133.4–135 ppm. The ^31^P-NMR data confirmed that the condensation of phenol with the lignin side chains has occurred, as evidenced by the content of *p*-Hydroxy phenyl OH increased from 0.012 mmol/g to 0.369 mmol/g. It also could be observed that the content of Syringyl OH, Guaiacyl OH in lignin decreased after phenolation, which was owing to the demethoxy reaction during phenolation^30^. This led to the reduction of S-type and G-type which contained more than one methoxyl group on its aromatic ring, reducing the substitution on benzene rings and the steric hindrance. These results were consistent with H-NMR data. Meanwhile, the aliphatic OH content of PPAL was 0.139 mmol/g, which was higher than that in PAL, indicating that phenolation could increase the content of aliphatic hydroxyl groups.

**Table 1:** Quantification of the functional groups (mmol/g) in the lignins of PAL and PPAL using a quantitative 31P-NMR method.

δ(ppm)	Assignment	PAL (mmol/g)	PPAL (mmol/g)
145–148	Aliphatic OH	0.072	0.139
141.5–143	Syringyl OH	0.368	0.056
138.5–139.5	Guaiacyl OH	0.180	0.060
137–137.9	*p*-Hydroxy phenyl OH	0.012	0.369
133.4–135	Carboxylic OH	0.024	0.004
	Active sites^a^	0.192	0.429

^a^The amount of active sites available for the subsequent graft polymerization; Active sites = Guaiacyl OH + *p*-Hydroxy phenyl OH.

### Synthesis and characterization of lignin-based gel

As the final aim of this work, PPAL-based gel was synthesized with PPAL and NIPAAm. In the compound triggering system, hydrogen peroxide and chloride ions form a peroxyl radical and chlorine atom. Activated chlorine atom reacts with a hydrogen atom from lignin and forms a grafting site on that backbone^31^. These hydrogen atoms were mostly provided by phenolic hydroxyl group for its high activity. The lignin radical was then assumed to react with a NIPAAm to start graft copolymerization (as illustrated in Fig. 1). The properties of the obtained gels were also characterized.

**Figure 6:**
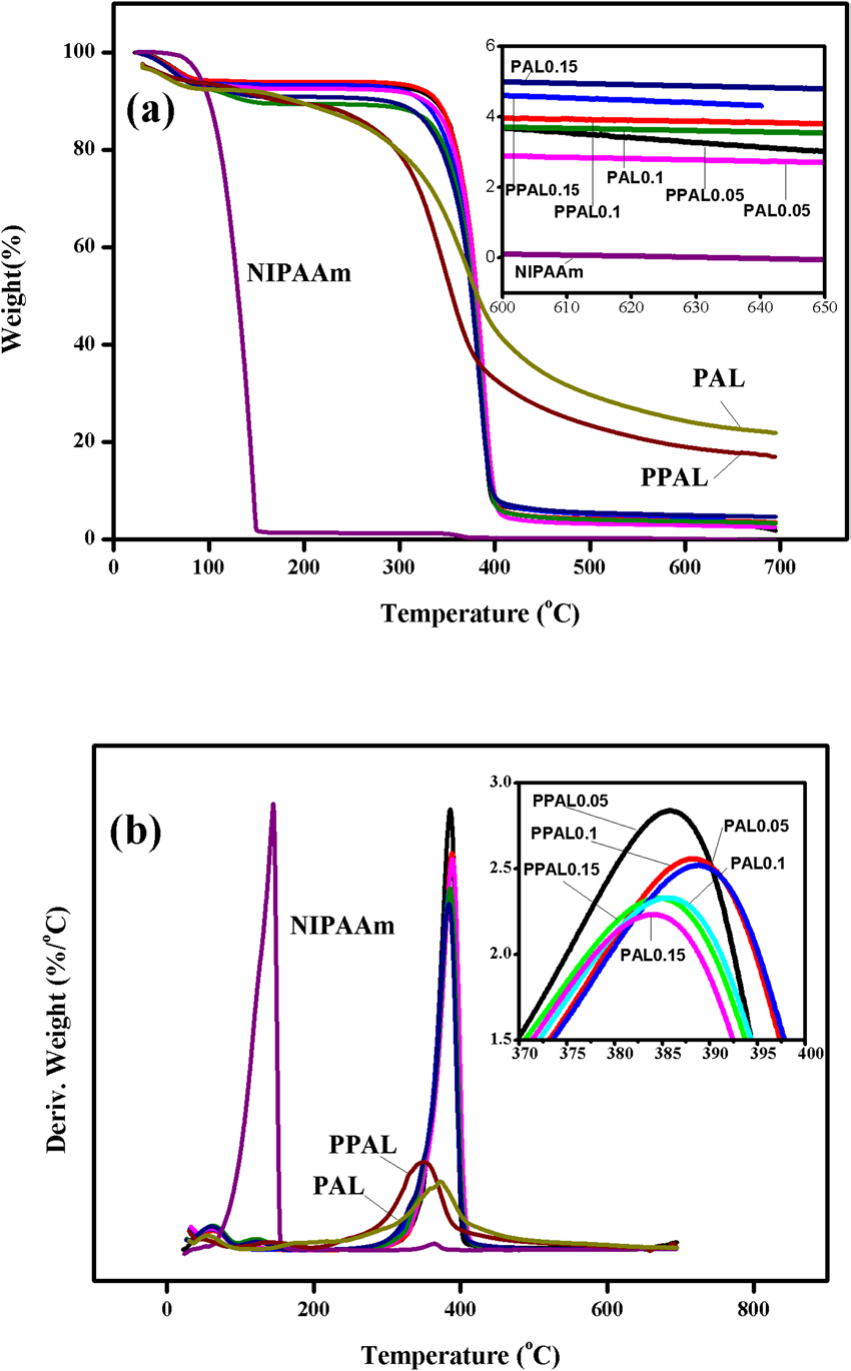
TG and DTG curves of lignin and gels. (**a**) TG curve; (**b**) DTG curve.

Thermal stability is one of the important properties of polymer. The thermal stability of lignin, pure NIPAAm, PAL-gel, and PPAL-gel has been characterized by TGA (as shown in Fig. 6). After modification with phenol, PPAL exhibited lower temperature at 50% weight loss (382.9 °C) than that of PAL (432.5 °C). Besides, the decrease of DTG maxima was also observed (PAL 373.8 °C, PPAL 350.3 °C), this trend was also suitable for residue content (PAL 38.6%, PPAL 34.5%). The result indicating that the thermo stability of lignin was reduced. This may be due to the higher hydroxyl content in PPAL, which volatilizes at a relatively low temperature. It can be observed that pure NIPAAm has a lower temperature (130.7 °C) at 50% weight loss than that of lignin-based gels (ranged from 375.7 °C to 381.3 °C), indicating that the thermal stability is improved after introduction of lignin into NIPAAm. This may attribute to the formation of highly condensed aromatic structures in lignin^32^. As shows in Table 2, the DTG maxima of pure NIPAAm was 145 °C while lignin-based gels exhibited higher DTG maxima above 380 °C at which pure NIPAAm was totally degraded. Previous report indicated that lignin molecular structure was composed of mostly aromatic rings with various branching, these chemical bonds led to a wide range of degradation temperature from 100 to 800 °C and about 30 to 40 wt% still remained un-volatized above 800 °C^33^. Lignin-based gels have formed char residue after being heated from 30 °C to 700 °C at a rate of 10 °C/min, this might attribute to the addition of lignin and the mass of char residue increased with the increment of lignin content. This result was consistent with the previous research^34^.

**Table 2:** Thermal properties of lignin-based gels and NIPAAm.

Sample	Temperature at 50% weight loss (°C)	DTG maxima (°C)	Char residue (wt%)
PAL	432.5	373.8	38.6
PPAL	382.9	350.3	34.5
NIPAAm	130.7	145	0
PAL0.05	381.0	388.6	2.8
PAL0.1	377.1	385.7	3.6
PAL0.15	375.7	384.1	4.8
PPAL0.05	379.4	386.0	3.0
PPAL0.1	381.3	388.4	3.8
PPAL0.15	377.5	384.6	4.3

**Figure 7:**
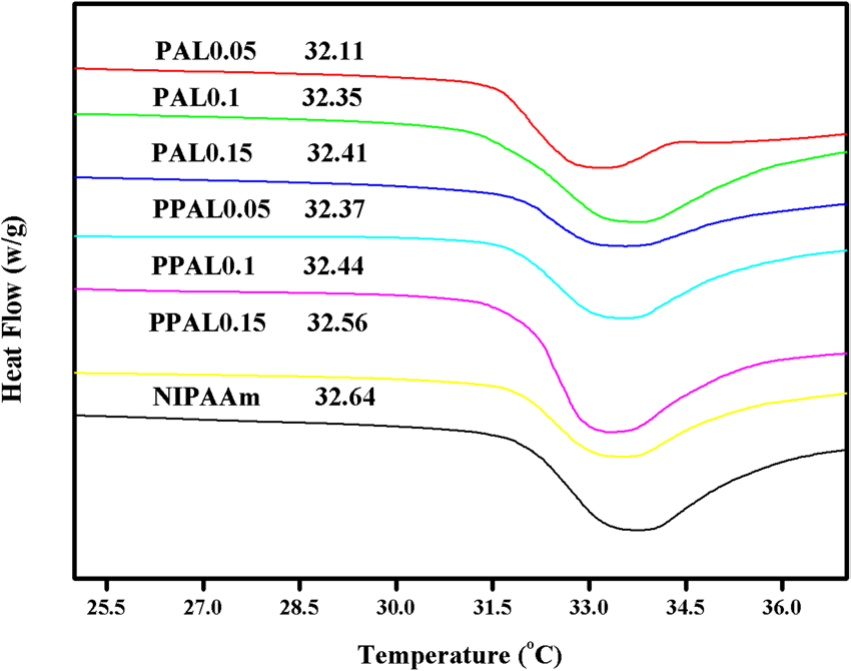
LCST of lignin-based gels and NIPAAm.

Lower Critical Solution Temperature (LCST) of lignin-based gel was characterized by DSC (as shown in Fig. 7). LCST of all lignin-based gels were lower than that of pure NIPAAm and decreased with the increasing lignin content in gels. Similar results were also found in previous research^3^. As expected, the LCST of PPAL-based gel was higher than PAL-based gel with the same amount of lignin. As we know, there exists a hydrophilic/hydrophobic equilibrium in NIPAAm, hydrophilic amide groups would interact with water through hydrogen bonds while the temperature was below the LCST, leading to the swelling of PNIPAAm. However, the PNIPAAm will become hydrophobic due to the dehydration when the temperature was above the LCST. The LCST increased with the increment of side groups’ hydrophilicity in NIPAAm. The introduction of hydrophobic lignin reduced the LCST of NIPAAm. Compared with PAL, the introduction of PPAL enhanced hydrophilicity of NIPAAm due to higher hydroxyl group content and reduced the space volume of hydrophobic side group grafted on each NIPAAm chain, which can explain that PPAL-gel has a higher LCST than PAL-gel.

**Figure 8:**
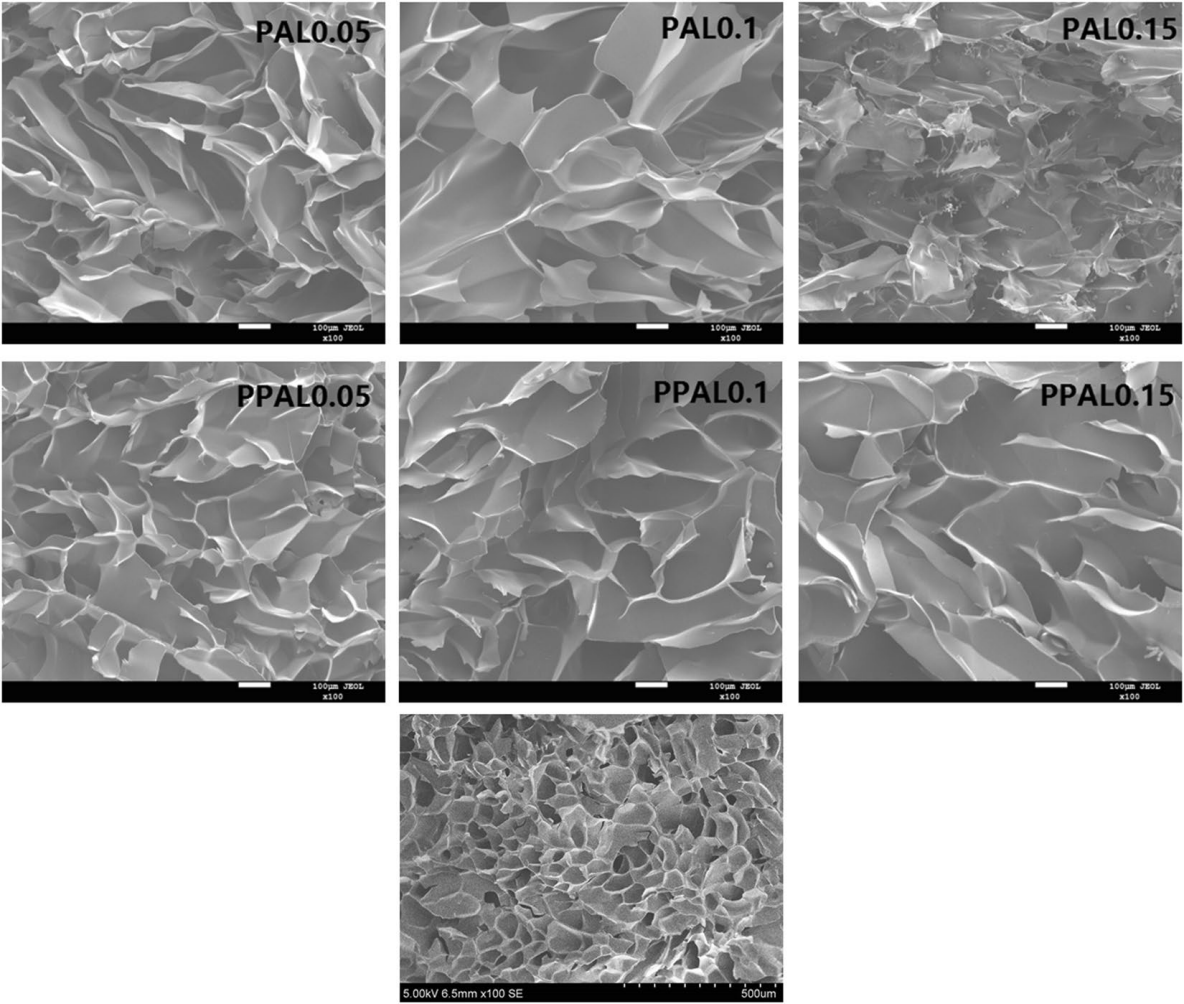
The SEM images of lignin-based gels.

Figure 8 demonstrated the interior morphology of lignin-based gels by SEM. Its mechanical properties were also provided in Table 3. The SEM images clearly illustrated that the pore of the pure NIPAAm was very dense and small, the introduction of lignin increase the pore size of gel and the macro-porosity was promoted with the increasing of lignin dosage in the gel. This result can be rationalized that the introduction of lignin into the gel system as an orifice agent can help improve pore diameter. However, excessive lignin will make the gel more brittle, which can be confirmed by the mechanical properties of the gel. The pore size was also effected by the crosslinking density^3^, the increasing crosslinking density will reduce the pore size and improve the mechanical properties, regularity. As shown in Table 3, PPAL-based gel exhibits better compressive strength compared with PAL-based gel under the same lignin content due to its higher crosslinking density. Therefore, pore size of gels could be controlled by changing the amount of lignin and crosslinking density, even to altering the gel network structure. It can be used to selectively concentrate and separate the specific substances.

**Table 3:** Compressive strength of lignin-based gels.

Sample	Compressive strength (KPa)
PAL0.05	8.82
PAL0.1	4.98
PAL0.15	0.71
PPAL0.05	11.15
PPAL0.1	9.55
PPAL0.15	5.66

**Figure 9:**
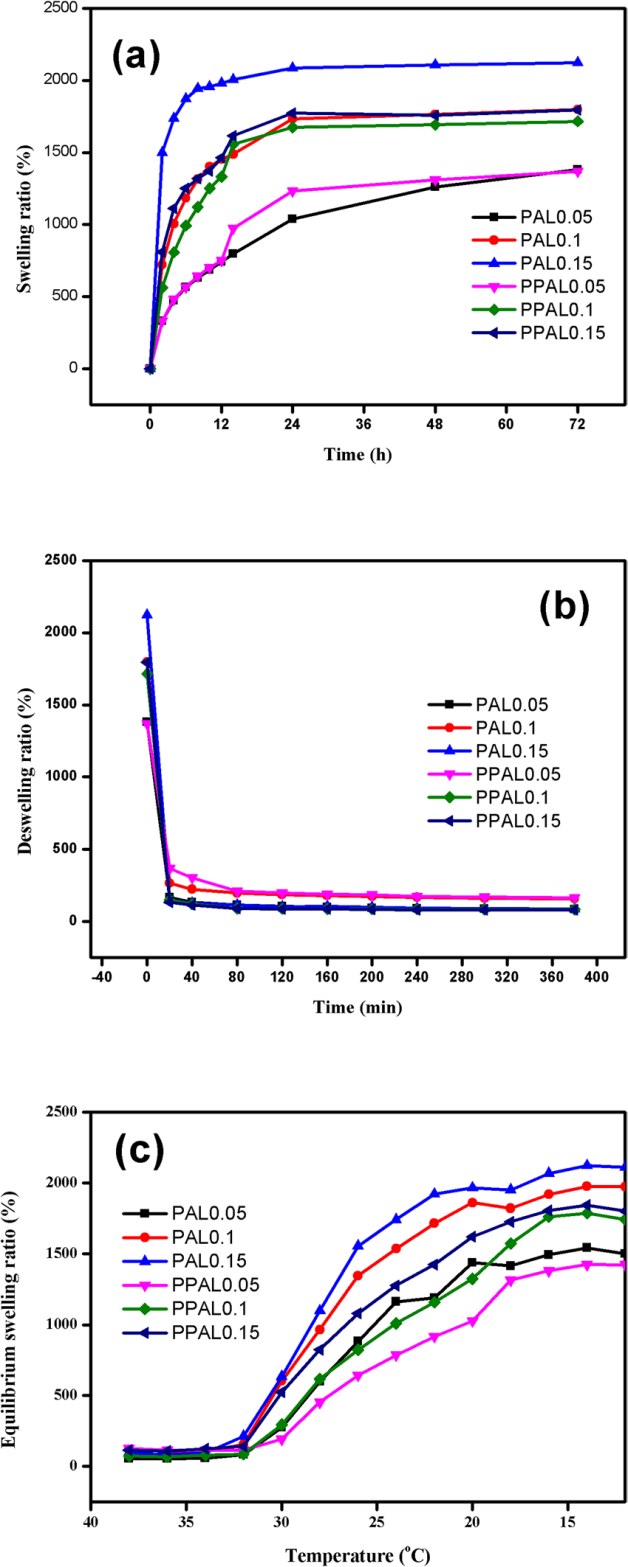
The swelling behavior, deswelling behavior. (**a**) swelling ratio at 20 °C; (**b**) deswelling ratio at 40 °C; (**c**) swelling ratio in the temperature range from 40 °C to 12 °C.

To investigate the swelling and deswelling kinetics of lignin-based gels, freeze-dried gels were immersed in deionized water until reaching its equilibrium state at 20 °C and 40 °C respectively. As shown in Fig. 9(a), all the gels have reached the maximum water absorption within 24 h at 20 °C and no significant effect after that. The curve also clearly illustrated the swelling ratio was affected by the lignin content and crosslinking density. PAL0.15 and PPAL0.15 exhibited the highest swelling ratio about 2125%, 1806%, while PAL0.05 and PPAL0.05 exhibited the lowest swelling ration about 1380.4%, 1369.5%, respectively. Overall, the PAL-based gel showed a higher swelling ratio and swelling rate under the same lignin dosage. This tendency might attribute to two possible reasons. Firstly, the increase of pore diameter, illustrated in interior morphology analysis, increases the adsorption capacity and the accessibility to water, which makes it easier for the water molecules entering into the gel and achieving the equilibrium state. In addition, the higher crosslinking level of PPAL-based gel shows more complex structure and lower pore size, makes it more difficult for water entering into the gel, reducing the swelling rate and lowering the adsorption capacity.

The deswelling behavior of gels was also showed in Fig. 9(b). It can be observed that all the gels exhibited relatively fast response rates at 40 °C, its swelling ratio dropped below 400% within 20 min and reached equilibrium state at 80 min, implying that the synthetic lignin-based gel shows good thermal-sensitive properties. There is no significant difference in the residual water content between PAL-based gel and PPAL-based gel, which means that the PPAL-based gel does not affect the release of adsorbate, while improving mechanical properties.

Figure 9(c) illustrates the equilibrium swelling ratio of lignin-based gel at the temperature range from 40 °C to 12 °C with an interval of 2 °C for 24 h. It was found that the equilibrium swelling ratio had an obvious change when temperature dropped to 32 °C. While the equilibrium swelling ratio of PAL0.15 and PPAL0.15 varied from 95%, 124% at 34 °C to 635%, 524% at 30 °C. The similar trend was also observed in PAL0.05, PPAL0.05 that swelling ration increased from 57%, 114% to 274%, 192%, respectively. Above information indicates that hydrogels turned from hydrophobic to hydrophilic at 32 °C. This was consistent with DSC result.

This thermo-sensitive property can be applied in the field of pest and disease prevention and control. It can realize the independent release and recovery of chemicals through the temperature change of nature. In addition, lignin has the function of absorbing ultraviolet light. This property has a good preservation effect for UV decomposable drugs.

## Conclusion

A novel lignin-based thermo-sensitive gel was synthesized with phenolated alkali lignin and NIPAAm through thermal polymerization. Compared with PAL-based gel, PPAL-based gel exhibited a higher crosslinking density and LCST, more regular interior morphology and better mechanical properties. Furthermore, the highest swelling ratio of PPAL-based gel (PPAL0.15, 1806%) was lower than that of PAL-based gel (PAL0.15, 2125%) due to its more complex structure.

## Methods

### Purification of alkali lignin (PAL)

300.0 g alkali lignin (AL) was dissolved in 1000 ml deionized water with pH = 12, adjusted by sodium hydroxide solution (0.2 mol/L), filtered to remove insoluble impurities. Then adjusted pH of the solution to 2.0-3.0 by sulfuric acid (0.2 mol/L) and kept in water bath (70 °C) for 1 h. The precipitated AL was filtered and washed to neutral with deionized water. Finally, the purified alkali lignin (PAL) was obtained after freeze-dried.

### Preparation of phenolated lignin (PPAL)

Phenol and PAL (20 wt% of phenol) were blended in a flask. 10% of phenol weight sulfuric acid (98 wt%) was added into the solution, the flask was placed into water bath (90 °C) for 2 h with rapid stirring. Then, the solution was washed with saturated NaCl until aqueous layer turned neutral, phenolated lignin was precipitated from organic layer (upper layer, black) by diethyl ether, washed with diethyl ether (4 × 50 ml) and dried.

### Synthesis of lignin-based gel

The synthesis of lignin-based gel was carried out according to the previous literature with minor modification^19^. Briefly, PAL or PPAL (0.05 g, 0.10 g, 0.15 g) was dissolved in 2 ml dimethyl sulfoxide (DMSO) with 0.2 g calcium chloride powder. Then hydrogen peroxide (5 wt% of lignin), 1.2 g NIPAAm, 0.017 g N,N′-methylenebisacrylamide (MBA, the crosslinking agent) were added into the solution. After all the agents were stirred into homogeneous solutions, bubbled with N_2_ for 10 min and sealed to place into a 70 °C bath for 12 h. The gel was soaked in DMSO (or deionized water) for 5 days at room temperature, to remove unreacted lignin and other impurities. The prepared gels were named as PAL0.05/0.1/0.15 and PPAL0.05/0.1/0.15 respectively. DMSO (or deionized water) was replaced per 12 h. After that, the swelling hydrogel was used to DSC analysis prior to freeze- dry at −50 °C for 24 h, vacuum-dried at 40 °C for 12 h for TGA, SEM, FT-IR analysis.

### Analytical Methods

FT-IR spectra of PAL, PPAL, gels were characterized using a Frontier Spectrometer (PE, USA), in the range from 4000 cm^−1^ to 400 cm^−1^. Samples were grounded and pelletized with potassium bromide, which was dried at 105 °C over 12 h prior to experimental analysis.

Thermal stability was carried out on a TA Q500 thermal analyzer (TA Instruments, USA). The apparatus was continually purged with nitrogen at a flow of 40 mL/min, and then the sample was heated from 30 °C to 700 °C at a rate of 10 °C/min.

LCST of samples were measured using differential scanning calorimeter (DSC250, TA, USA). The apparatus was continually purged with nitrogen at a flow of 40 mL/min, and then samples were heated from 0 °C to 40 °C at a rate of 10 °C/min, referenced against an empty pan.

The molecular weight of lignin samples including weight average molecular weights (M_w_), number average molecular weight (M_n_), polydispersity were measured by GPC instrument (Waters 1515/2414, USA), which equipped with a refractive index detector and two cascaded PL-gel columns at 35 °C with tetrahydrofuran (THF) as the mobile phase at a rate of 1.0 mL/min. Calibration was performed using polystyrenes with a molecular mass range between 1150 Da and 598 kDa. 300 mg lignin samples were subjected to acetylation by dissolving in 15 ml acetic anhydride/pyridine (2:1, v/v) mixture and stored in a cool and ventilated environment under anaerobic atmosphere for 72 h. Acetylated lignin was precipitated and washed by diethyl ether (200 × 3 ml). Then, dried acetylated lignin was dissolved in THF (10 mg/ml), filtered for analysis.

NMR for PAL, PPAL was performed in a Bruker AVIII 400 MHz spectrometer. ^31^P-NMR analysis was according to a previous report with minor modification^35,36^. 25 mg lignin samples were dissolved in 0.5 ml anhydrous pyridine/CDCl_3_ solvent (1.6:1, v/v) under stirring, 0.1 ml internal standard solution including 10.25 mg/ml cyclohexanol in anhydrous pyridine/CDCl_3_ solvent (1.6:1, v/v) and 0.1 ml relaxation reagent solution containing 5.1 mg /ml chromium (III) acetylacetonate in anhydrous pyridine/CDCl_3_ solvent (1.6:1 v/v) was added. Successively, 0.1 ml 2-chloro-4,4,5,5-tetramethyl-1,3,2-dioxaphospholane (TMDP) was added into the mixture and stirred for 2 h. Finally, phosphitylated lignin samples were transferred into 5 mm NMR tube for analysis. For ^1^H-NMR, 15 mg acetylated lignin samples were weighed and dissolved in 0.5 ml DMSO-d_6,_ filtered prior to analysis.

Mechanical properties of gels were tested using universal material testing machine (INSTRON5960, USA) with a speed of 2 mm/min. All the samples were cut into 22.5 mm (radius) and 10 mm (height) after removal of the surface water with filter paper.

The interior morphology of the hydrogels was analyzed by a scanning electron microscope (SEM, JSM-7800F, Japan). All samples were coated with gold prior to analysis.

### Swelling/deswelling behavior and equilibrium swelling ratio

The swelling ratio of gels was measured by immersing freeze-dried gel in deionized water at 20 °C. Free water on the swollen hydrogels surface was removed by filter paper and the weight (W_t_) was recorded per 2 h. The swelling ratio was calculated as follow:

$${\rm{Swelling}}\,{\rm{ratio}}( \% )=({{\rm{W}}}_{{\rm{t}}}-{{\rm{W}}}_{{\rm{fd}}})/{{\rm{W}}}_{{\rm{fd}}}\times {\rm{100}} \% $$

W_fd_: the weight of freeze-dried gel.

After reaching equilibrium state at 20 °C, the swollen hydrogel was immersed at 40 °C for deswelling behavior characterization. The weight of hydrogel was recorded per 2 h. The deswelling ratio was calculated as follow:

$${\rm{Deswelling}}\,{\rm{ratio}}( \% )=({{\rm{W}}}_{{\rm{td}}}-{{\rm{W}}}_{{\rm{fd}}})/{{\rm{W}}}_{{\rm{fd}}}\times {\rm{100}} \% $$

W_td_: the weight of deswelled-gel.

Equilibrium swelling ratio of gels was measured by immersing freeze-dried gel in deionized water for 24 h at the temperature range from 40 °C to 20 °C with an interval of 2 °C. The weight of hydrogel was recorded as described above and the equilibrium swelling ratio was calculated as follow:

$${\rm{Equilibrium}}\,{\rm{swelling}}\,{\rm{ratio}}( \% )=({{\rm{W}}}_{{\rm{ES}}}-{{\rm{W}}}_{{\rm{fd}}})/{{\rm{W}}}_{{\rm{fd}}}\times {\rm{100}} \% $$

W_ES_: the weight of equilibrium swelled-gel.
